# Pareto-Guided Process Optimization of Single-Crystal InP Chemical Mechanical Polishing: Balancing Material Removal Rate and Surface Roughness

**DOI:** 10.3390/mi17101108

**Published:** 2026-09-22

**Authors:** Jiayun Deng, Junjiang Zhou, Jiacheng Geng, Hua Wei, Hanbao Liu, Jiao Li, Shunjin Wang, Changlin Li, Tinglong Liu, Feng Hui

**Affiliations:** 1 Yunnan Lincang Xinyuan Germanium Industry Co., Ltd., Lincang 677000, China; huifeng@sino-ge.com; 2 Faculty of Mechanical and Electrical Engineering, Kunming University of Science and Technology, Kunming 650500, China; zhoujunjiang@stu.kust.edu.cn (J.Z.); 17806903051@163.com (J.G.); 3 Yunnan Xinyao Semiconductor Materials Co., Ltd., Kunming 650503, China; weihua@sino-ge.com (H.W.); liuhanbao@sino-ge.com (H.L.); wangshunjin@sino-ge.com (S.W.); liutinglong@sino-ge.com (T.L.); 4 School of Metallurgy and Energy Engineering, Kunming University of Science and Technology, Kunming 650500, China; 5 Yunnan Zhongke Xinyuan Crystal Materials Co., Ltd., Kunming 650503, China; lijiao@sino-ge.com (J.L.); lichanglin@sino-ge.com (C.L.)

**Keywords:** single-crystal InP, chemical mechanical polishing, regression analysis, response surface methodology, multi-objective optimization

## Abstract

Single-crystal indium phosphide (InP) is an important substrate material for high-speed electronic and optoelectronic devices. Its relatively low hardness, brittleness, and sensitivity to polishing chemistry make it challenging to achieve both efficient material removal and low surface roughness during chemical mechanical polishing (CMP). In this study, response surface methodology (RSM) was combined with multi-objective particle swarm optimization and the Technique for Order Preference by Similarity to Ideal Solution (MOPSO–TOPSIS) to investigate the balance between material removal rate (MRR) and surface roughness (Ra). A three-factor Box–Behnken design was used to establish quadratic models incorporating H_2_O_2_ concentration, SiO_2_ abrasive concentration, and polishing speed. The models gave coefficients of determination of 0.9898 for MRR and 0.9894 for Ra. Abrasive concentration had the strongest influence on both responses within the investigated ranges. Higher abrasive concentrations favored material removal, whereas lower roughness was obtained at intermediate concentrations and polishing speeds. A compromise condition of 9.79 wt% H_2_O_2_, 1.08 wt% SiO_2_, and 54.33 rpm was evaluated experimentally. The predicted MRR and Ra were 3871.32 nm/h and 0.6747 nm, respectively, compared with measured values of 4283.79 nm/h and 0.712 nm. The corresponding relative deviations were 10.65% and 5.53%. These results provide a quantitative basis for selecting CMP parameters that balance removal efficiency and surface roughness in single-crystal InP polishing.

## 1. Introduction

Single-crystal indium phosphide (InP) is a III–V compound semiconductor with a direct bandgap, high electron mobility, and favorable high-frequency characteristics, making it an important material for high-speed electronic and optoelectronic devices [1]. The preparation of InP substrates requires effective control of the surface condition because defects introduced during processing can affect subsequent epitaxial growth and integration. However, the low hardness and brittleness of InP make its surface susceptible to mechanical damage, with deformation and fracture also influenced by crystal orientation [2,3]. Grinding and lapping are important stages of wafer preparation, and the resulting surface integrity affects subsequent polishing. Gao et al. [4] demonstrated this relationship for silicon wafers, showing that differences between ground and lapped surfaces influence both removal efficiency and surface improvement during CMP. For III–V substrates, the need to control roughness and polishing defects is also reflected in studies of wafer bonding and defect characterization [5,6].

Chemical mechanical polishing combines interfacial chemical reactions with mechanical removal to produce smooth wafer surfaces. Research on InP CMP has examined the effects of slurry composition, pH, abrasive properties, and processing conditions. Morisawa et al. [7,8] investigated both a colloidal silica–citric acid–H_2_O_2_ slurry and a NaOCl–citric acid system, demonstrating the importance of coordinating slurry chemistry with polishing conditions. Peddeti et al. [9] studied the influence of chemical and abrasive conditions on InP removal and surface quality. Matovu et al. [10] further showed that multifunctional carboxylic acids and H_2_O_2_ could improve the polished surface while reducing phosphine evolution. These studies established the importance of controlling chemical modification and mechanical action together.

More recent work has extended both the process and chemical aspects of InP polishing. Li et al. [11] examined the effects of processing parameters on the removal rate of InP wafers. Qi et al. [12] investigated EDTA and glycine as complexing agents for improving alkaline InP CMP, while Fu et al. [13] developed a hexafluorophosphate-assisted process involving fluoride-mediated surface modification. Our previous study [14] compared the oxidation and polishing behavior of InP under different oxidizing conditions and identified H_2_O_2_-based slurries as suitable for fine polishing. Although these studies provide useful guidance for slurry selection and individual parameter adjustment, determining a balanced combination of oxidant concentration, abrasive concentration, and polishing speed remains a practical requirement.

Experimental design and predictive modeling offer a means of relating these processing variables to polishing performance. Previous work has combined experimental design with nonlinear modeling for CMP optimization [15], used multi-response analysis to select semiconductor lapping conditions [16], and applied response surface methodology to chemically assisted polishing [17]. Physics-informed machine learning and residual convolutional neural networks have also been developed to predict MRR in CMP [18,19]. For an experimentally defined polishing system, a quadratic response model provides a direct way to examine the main effects, curvature, and pairwise interactions of the selected factors.

In H_2_O_2_–SiO_2_ CMP of InP, increasing chemical or mechanical action can improve removal efficiency without necessarily reducing roughness. Individual parameter trends therefore do not, by themselves, determine the most suitable combined operating condition. Building on our previous work [14], this study uses a Box–Behnken design to establish response models for MRR and Ra as functions of H_2_O_2_ concentration, SiO_2_ concentration, and polishing speed. Analysis of variance and surface topography are used to examine the polishing response, and MOPSO–TOPSIS is employed to select a compromise condition for experimental verification. The objective is to connect the coupled parameter effects with practical selection of polishing conditions that balance efficiency and roughness.

## 2. Experiments

### 2.1. Experimental Setup and Principle

The CMP experiments were performed using a KD18BX grinding and polishing machine (KIZI, Dongguan, China). Figure 1 shows the apparatus and the polishing configuration. The system consists of a rotating platen, polishing pad, wafer–carrier assembly, loading components, and slurry supply unit. The wafer was attached to a ceramic carrier using paraffin wax and pressed against the polishing pad under a prescribed load. Platen rotation generated relative motion at the polishing interface, while a peristaltic pump continuously supplied slurry.

Material removal involves chemical modification of the InP surface followed by removal through abrasive and pad contact. Studies of InP oxidation and hydroxylation show that surface structure and bonding influence its interfacial chemical behavior [20,21]. In the H_2_O_2_-containing slurry, representative overall oxidation reactions can be written in molecular form as Equations (1) and (2) [10,14]:

(1) $${\mathrm{InP}(s)\;+\;2H}_{2}O_{2}(\mathrm{aq})\;\to \;\frac{1}{2}{\mathrm{In}}_{2}O_{3}(s)+H_{3}{\mathrm{PO}}_{2}(\mathrm{aq})+\frac{1}{2}H_{2}O(l)$$

(2) $${\mathrm{InP}(s)+2H}_{2}O_{2}{(\mathrm{aq})+H}_{2}O(l)\to {\;\mathrm{In}(\mathrm{OH})}_{3}(s)+H_{3}{\mathrm{PO}}_{2}(\mathrm{aq})$$

The chemically modified surface is subjected to repeated contact with SiO_2_ particles and the polishing pad. Removal of this material exposes fresh InP to the slurry, allowing surface reaction and mechanical removal to continue. This description is consistent with CMP models that account for the formation and removal of a chemically altered surface layer [22].

### 2.2. Experimental Materials and Design

Single-crystal InP wafers with a diameter of 50.8 mm and a (100) surface orientation were supplied by a semiconductor company in Yunnan, China. The polishing slurry contained H_2_O_2_ as the oxidant and colloidal SiO_2_ with a nominal mean particle diameter of 20 nm as the abrasive. Polyethylene glycol was added at 0.25 wt% as a dispersant. The slurry pH was adjusted to 7 using citric acid and sodium hydroxide.

A black damping polishing pad was used. The polishing pressure was fixed at 0.037 MPa based on preliminary experiments. The slurry flow rate and polishing duration were maintained at 30 mL/min and 40 min, respectively.

The ranges of H_2_O_2_ concentration, SiO_2_ concentration, and polishing speed were selected on the basis of our previous InP CMP study and the associated single-factor experiments [14]. These variables were designated *A*, *B*, and *C*, respectively. A three-factor, three-level Box–Behnken design [23] was generated using Design-Expert software (version 13.0, Stat-Ease, Inc., Minneapolis, MN, USA). The design comprised 12 noncentral runs and three center-point runs, with the factor levels listed in Table 1.

Complete quadratic models containing linear, squared, and pairwise interaction terms were fitted to MRR and Ra by least squares. ANOVA was used to evaluate the models and individual terms.

After polishing, the wafers were cleaned ultrasonically with a dedicated cleaning agent, followed by cleaning in deionized water and absolute ethanol for 10 min each. The samples were then dried before measurement.

### 2.3. Characterization

Surface roughness and topography were measured using a SuperView WM100 optical 3D surface profiler (CHOTEST, Shenzhen, China). Measurements were taken at the wafer center and at four symmetrically distributed locations positioned 4 mm inward from the wafer edge. The average Ra of these five locations was used to characterize each wafer. The standard deviation among the locations described the spatial variation in the roughness measurements.

Wafer mass was measured before and after polishing using an AX224ZH analytical balance (OHAUS, Shanghai, China; readability: 0.1 mg). MRR was calculated from the mass loss using Equation (3):

(3) $$\mathrm{MRR}=\frac{\Delta m*10^{7}}{{\rho \pi r}^{2}t}$$

where *Δm* is the wafer mass loss in grams, *ρ* is the InP density of 4.81 g/cm^3^, *r* is the wafer radius of 2.54 cm, and *t* is the polishing duration in hours. The factor 10^7^ converts the removed thickness from centimeters to nanometers, giving MRR in nm/h.

MRR characterizes the rate of thickness removal and therefore the processing time required for a given removal allowance, while Ra characterizes the resulting surface roughness. The two responses were selected to evaluate polishing efficiency and surface finish.

### 2.4. Experimental Results

Table 2 presents the experimental conditions and measured responses.

The measured MRR ranged from 1171.11 to 5732.27 nm/h, and Ra ranged from 0.452 to 1.218 nm. Run 1 produced the highest measured MRR and the highest Ra, whereas the lowest measured Ra occurred in Run 9 at the center-point condition. These results indicate that the processing conditions favoring high removal efficiency differ from those favoring a smooth surface.

## 3. Regression Model Analysis

The quadratic models were fitted using coded factors and are expressed below in the actual variables *A*, *B*, and *C*. *A* and *B* denote the numerical concentrations in wt%, and *C* denotes polishing speed in rpm.

### 3.1. MRR Response

The quadratic model for MRR was obtained as Equation (4):

(4) $$\begin{array}{l} \mathrm{MRR}=-2734.65-17.20\times A-5155.13\times B+187.03\times C+0.494\times A^{2}+4564.27\times B^{2}\\ -1.84\times C^{2}+72.42\times \mathrm{AB}+0.23\times \mathrm{AC}+23.11\times \mathrm{BC} \end{array}$$

The predicted response is expressed in nm/h. The corresponding ANOVA results are listed in Table 3.

The overall model was significant, with an *F*-value of 54.1398 and a *p*-value of 0.0002. The lack-of-fit test was not significant relative to the center-point pure error. The *R*^2^ and adjusted *R*^2^ values were 0.9898 and 0.9716, respectively, indicating that the model captured most of the measured variation.

Among the linear terms, abrasive concentration had the largest *F*-value, followed by H_2_O_2_ concentration and polishing speed. The terms *A*, *B*, and *B*^2^ were significant at the 0.05 level. The polishing-speed term had a *p*-value of 0.0550, and none of the pairwise interactions reached the 0.05 significance level. The most pronounced nonlinear dependence of MRR was therefore associated with abrasive concentration.

Figure 2a,b show that MRR increases with increasing H_2_O_2_ concentration and abrasive concentration, and the slope of the response surface along the abrasive concentration direction is significantly greater than that along the H_2_O_2_ concentration direction, indicating that abrasive concentration plays a dominant role in affecting MRR. This is consistent with the ranking of factor importance obtained from the regression model.

Figure 2c,d show that MRR generally increases with H_2_O_2_ concentration and exhibits a curved response to speed. The gain associated with increasing speed becomes less pronounced toward the upper part of the investigated range. 

In Figure 2e,f, changing abrasive concentration produces a substantially larger variation in MRR than changing speed alone.

The combined trends can be understood in terms of the availability of mechanically active contacts and the susceptibility of the modified surface to removal. CMP models based on weakly bonded surface species and chemical–mechanical synergy provide a basis for this interpretation [24,25,26]. Within the present experimental domain, the data support a strong concentration-dependent removal response, while the ANOVA results do not establish a distinct critical abrasive concentration.

### 3.2. Surface Roughness Response

The fitted Ra model is expressed by Equation (5):

(5) $$\begin{array}{l} \mathrm{Ra}=5.606-0.1767\times A-1.9385\times B-0.1362\times C+0.00802\times A^{2}+1.2127\times B^{2}\\ +0.00123\times C^{2}+0.0209\times \mathrm{AB}+0.0002\times \mathrm{AC}+0.00245\times \mathrm{BC} \end{array}$$

The predicted Ra is expressed in nm. Table 4 presents the ANOVA results.

The Ra model was significant, with an F-value of 51.8118 and a *p*-value of 0.0002. The lack-of-fit *p*-value was 0.4781. The *R*^2^ and adjusted *R*^2^ values were 0.9894 and 0.9703, respectively. The fitted response captures both the low-roughness region near the center of the experimental domain and the increase toward less favorable parameter combinations.

All three linear terms and all three quadratic terms were significant at the 0.05 level. Among the linear terms, the effects were ranked as *B* > *C* > *A*. The *AB* interaction was also significant, with a *p*-value of 0.0405, indicating that the effect of abrasive concentration on Ra depends partly on H_2_O_2_ concentration.

Figure 3a,b show a low-roughness region at moderate oxidant and abrasive concentrations. Moving away from this region increases Ra. This response reflects the need to coordinate chemical modification with mechanical removal: sufficient abrasive action is required to remove surface irregularities, but stronger mechanical action can also introduce or deepen polishing grooves. The dependence on H_2_O_2_ concentration indicates that changing the chemical contribution alters the abrasive conditions required for a smooth surface, consistent with previous InP CMP studies [9,10,14].

Figure 3c,d show that low Ra is obtained at moderate H_2_O_2_ concentration and a polishing speed of approximately 50–55 rpm. Figure 3e,f show a similar low-roughness region at moderate abrasive concentration and intermediate speed. The significant squared terms account for the curvature of these responses, whereas the *AC* and *BC* interaction terms were not statistically significant.

Representative surface topographies from the preliminary abrasive-concentration experiments are presented in Figure 4. The initial surface had an Ra of 2.092 nm. Polishing with 0.50 wt% SiO_2_ reduced Ra to 0.387 nm, with an MRR of 1249.87 nm/h. At 1.00 wt% SiO_2_, MRR increased to 5523.92 nm/h, while Ra increased to 0.848 nm and directional grooves became more evident. At 1.25 wt% SiO_2_, the surface exhibited more pronounced intersecting grooves and an Ra of 1.222 nm. These observations illustrate how increasing abrasive concentration can improve removal efficiency while producing a rougher surface, providing morphological context for the competing responses in the regression analysis.

## 4. Multi-Objective Optimization

The response models identify different parameter regions for high MRR and low Ra. Higher abrasive concentrations favor removal efficiency, whereas lower roughness is obtained within a moderate concentration range. The models in Equations (4) and (5) were therefore used together in the optimization problem defined by Equation (6):

(6) $$\begin{array}{l} \mathrm{Minimize}\;f\left(x\right)=\min\left\{-\mathrm{MRR}\left(x\right)\right.,\left.\mathrm{Ra}\left(x\right)\right\}\\ x=\left\{A,B,C\right\}\\ s.t.\\ \mathrm{MRR}\geq 0\\ \mathrm{Ra}\geq 0\\ 5\leq A\leq 15\\ 0.25\leq B\leq 1.25\\ 40\leq C\leq 60 \end{array}$$

The negative sign converts MRR maximization into a minimization objective. The parameter bounds correspond to the experimental domain.

### 4.1. MOPSO Implementation

MOPSO extends particle swarm optimization by using nondominated solutions to guide the search across competing objectives [26,27]. In this study, each particle represented a combination of *A*, *B*, and *C*, and an external repository stored the nondominated solutions. The response models supplied the objective values during the search.

The algorithm was implemented in MATLAB R2024b (MathWorks, Natick, MA, USA). The population size, maximum number of iterations, and repository capacity were set to 300, 200, and 100, respectively. The personal and leader acceleration coefficients were 1.2 and 1.1, and the inertia weight was 0.99. Candidate solutions were restricted to the bounds in Equation (6). TOPSIS was then used to evaluate the resulting alternatives, following the combined optimization and decision-making approach used in previous studies [28,29].

### 4.2. TOPSIS Evaluation

TOPSIS evaluates alternatives according to their distances from positive and negative ideal solutions [30,31]. MRR was treated as a benefit criterion and Ra as a cost criterion, with equal weights assigned to the two responses.

For *m* candidate solutions, *y*_*i*1_ and *y*_*i*2_ denote the predicted MRR and Ra, respectively, of candidate *i*. The weighted normalized responses were calculated using Equation (7):

(7) $$u_{\mathrm{ij}}=w_{j}\frac{y_{\mathrm{ij}}}{\sqrt{\sum_{i=1}^{m}y_{\mathrm{ij}}^{2}}},j=1,2,w_{1}=w_{2}=0.5$$

The positive ideal solution comprises the maximum weighted normalized MRR and the minimum weighted normalized Ra. The negative ideal solution comprises the minimum weighted normalized MRR and the maximum weighted normalized Ra. The corresponding distances were calculated using Equation (8):

(8) $$S_{i}^{\pm }=\sqrt{\sum_{j=1}^{2}{\left(u_{ij}-u_{j}^{\pm }\right)}^{2}}$$

Here, *u_j_*^+^ and *u_j_*^−^ are the positive- and negative-ideal values for criterion *j*, respectively. The relative closeness coefficient was then calculated using Equation (9):

(9) $$D_{i}=\frac{S_{i}^{-}}{S_{i}^{+}+S_{i}^{-}},0\leq D_{i}\leq 1$$

A larger relative closeness coefficient indicates a more favorable balance under the specified weighting and candidate set.

### 4.3. Selected Polishing Condition

Figure 5 illustrates the predicted relationship between MRR and Ra for the candidate solutions. Increasing the removal rate generally entails an increase in roughness, so parameter selection requires a compromise between the two responses.

A condition of 9.79 wt% H_2_O_2_, 1.08 wt% SiO_2_, and 54.33 rpm was selected for experimental verification. Substitution into Equations (4) and (5), using the unrounded coefficients, gives a predicted MRR of 3871.32 nm/h and an Ra of 0.6747 nm.

Table 5 compares the predicted responses at the selected condition with the measured extremes among the design runs. The selected condition is predicted to provide a higher MRR than the lowest-Ra run while maintaining a lower Ra than the highest-MRR run. It therefore represents an intermediate operating condition for balancing removal efficiency and roughness.

### 4.4. Experimental Verification

The selected condition was evaluated while maintaining the other polishing parameters described in Section 2.2. As summarized in Table 6, the measured MRR and Ra were 4283.79 nm/h and 0.712 nm, respectively.

Both measured responses were higher than the corresponding predictions. The models describe the fitted response to the three selected variables, whereas individual polishing runs can also be affected by variations in the initial surface condition and wafer–pad contact. A greater effective mechanical contribution can increase both material removal and roughness, providing a plausible explanation for the common direction of the deviations. The verification experiment achieved an MRR above 4000 nm/h while maintaining subnanometer Ra, supporting the practical suitability of the selected condition for the investigated polishing system.

## 5. Conclusions

The effects of H_2_O_2_ concentration, SiO_2_ concentration, and polishing speed on single-crystal InP CMP were investigated using response surface modeling and multi-objective optimization. The main conclusions are as follows:

(1) Quadratic models described the experimental MRR and Ra responses with R^2^ values of 0.9898 and 0.9894, respectively. Abrasive concentration had the strongest influence on both responses. Its interaction with H_2_O_2_ concentration significantly affected Ra, whereas the dominant nonlinear dependence of MRR was associated with abrasive concentration.
(2) The parameter regions favoring high removal efficiency differed from those favoring low roughness. Higher abrasive concentrations substantially increased MRR, while lower Ra was obtained within a moderate parameter range. Representative surface topographies showed that stronger removal at increased abrasive concentration was accompanied by more pronounced polishing grooves.
(3) A compromise condition of 9.79 wt% H_2_O_2_, 1.08 wt% SiO_2_, and 54.33 rpm was selected. The predicted MRR and Ra were 3871.32 nm/h and 0.6747 nm, respectively. Experimental verification yielded 4283.79 nm/h and 0.712 nm, with relative deviations of 10.65% and 5.53%. These results provide a quantitative basis for selecting InP CMP conditions that balance removal efficiency and surface roughness.

## Figures and Tables

**Figure 1:**
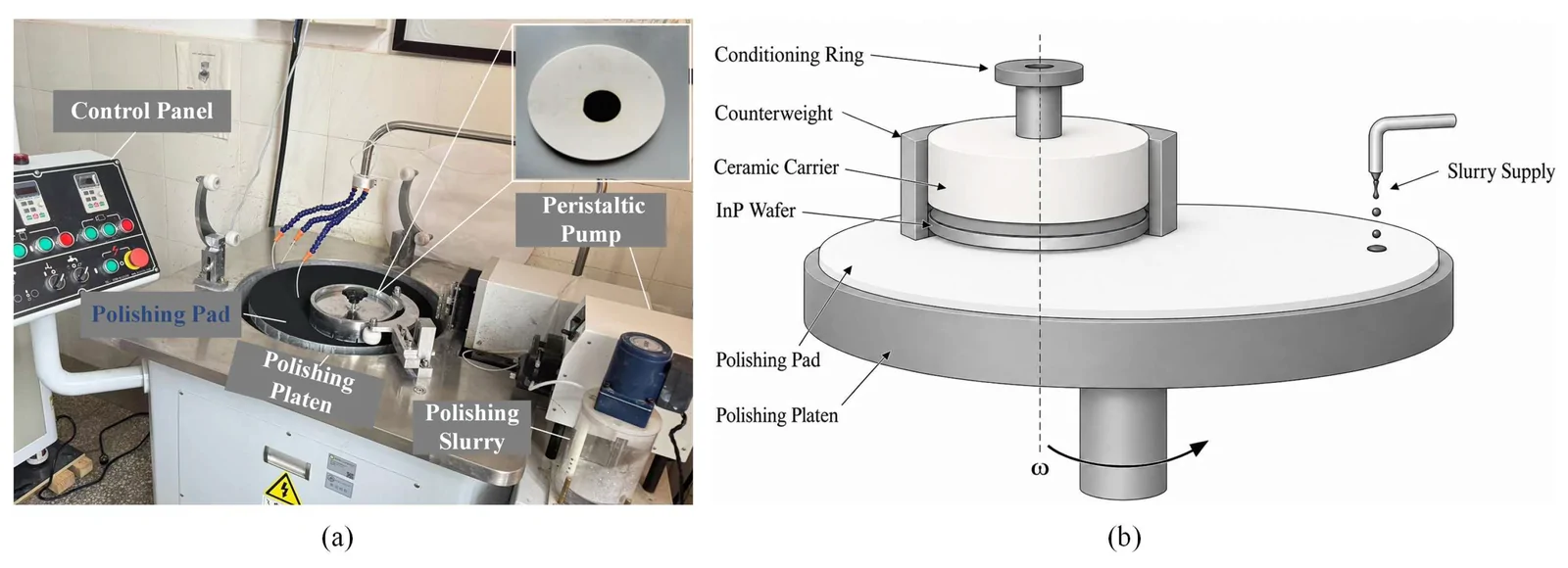
InP CMP: (**a**) experimental apparatus; (**b**) polishing configuration.

**Figure 2:**
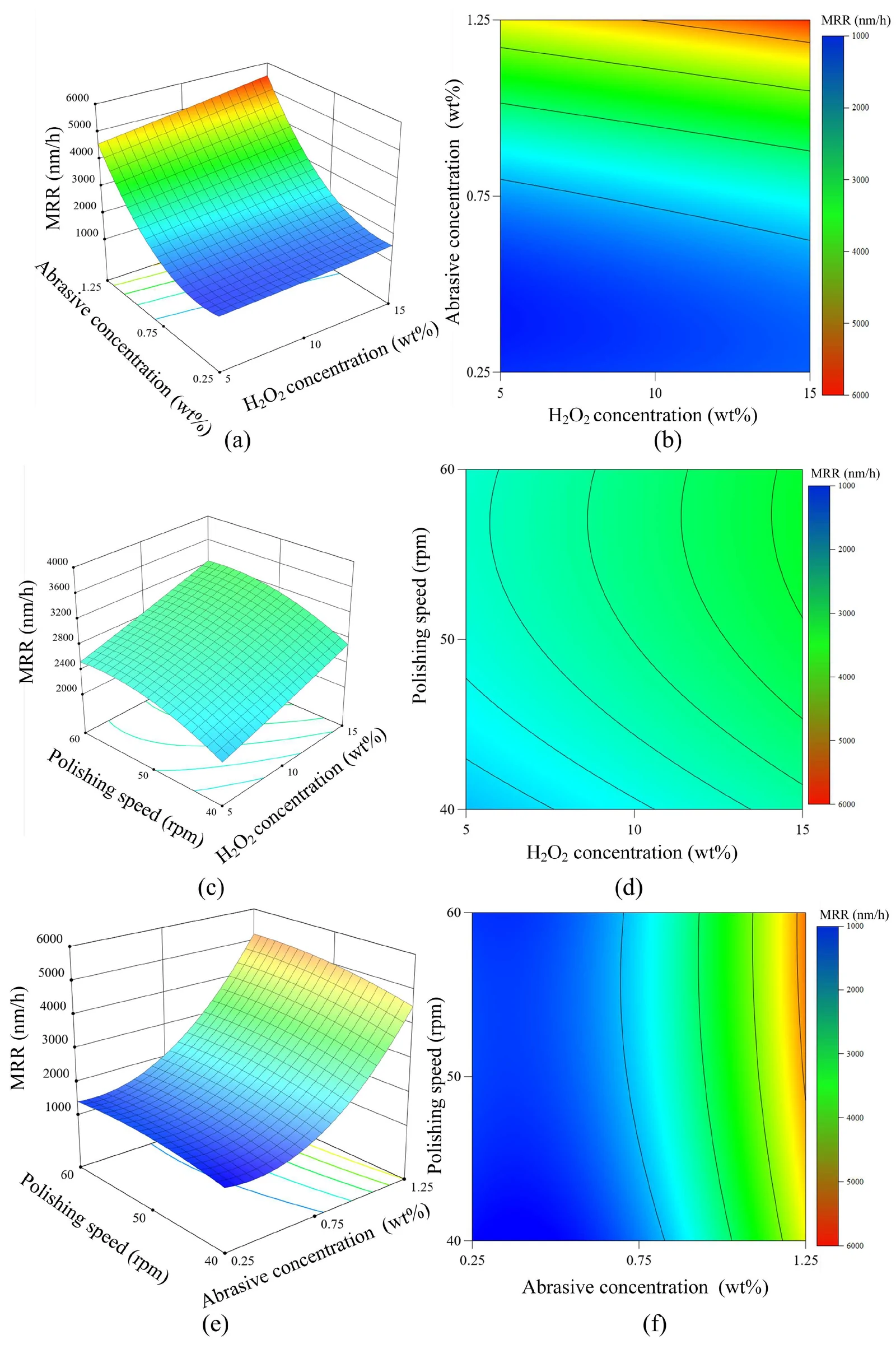
Effects of H_2_O_2_ concentration, SiO_2_ concentration, and polishing speed on MRR: (**a**,**c**,**e**) response surfaces; (**b**,**d**,**f**) contour plots.

**Figure 3:**
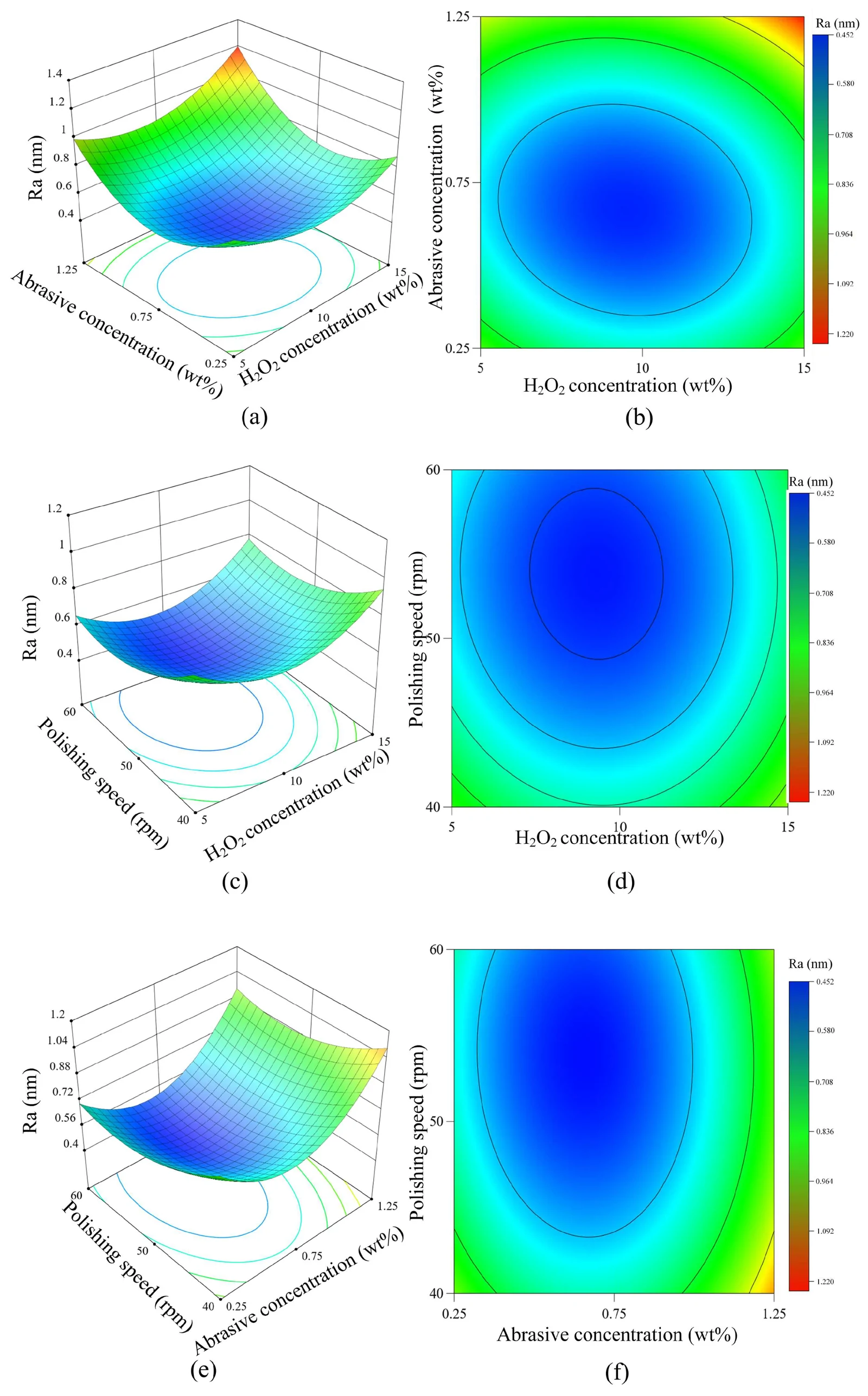
Effects of H_2_O_2_ concentration, SiO_2_ concentration, and polishing speed on Ra: (**a**,**c**,**e**) response surfaces; (**b**,**d**,**f**) contour plots.

**Figure 4:**
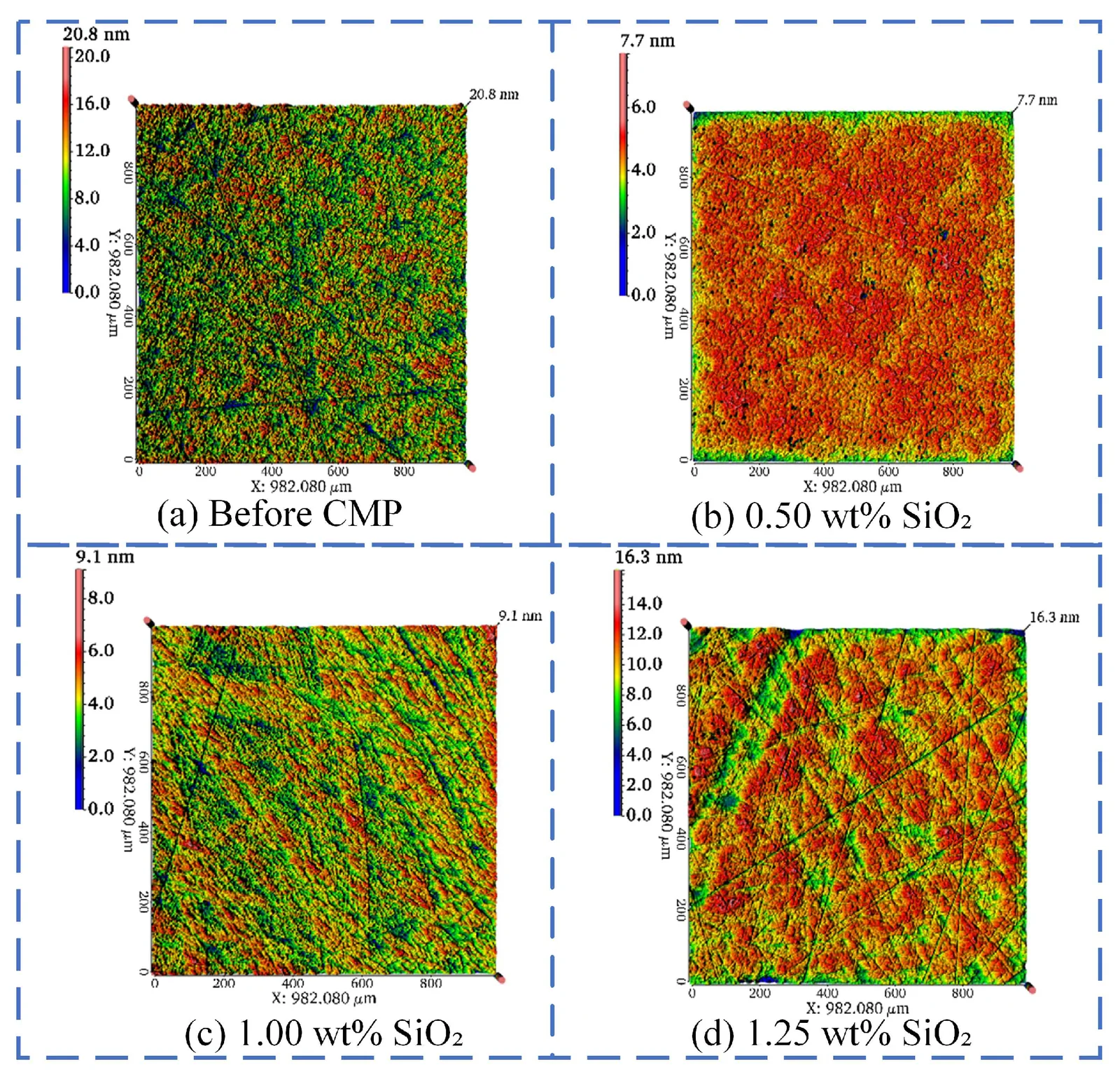
Surface topographies of InP wafers: (**a**) before CMP; after CMP with SiO_2_ concentrations of (**b**) 0.50 wt%, (**c**) 1.00 wt%, and (**d**) 1.25 wt%.

**Figure 5:**
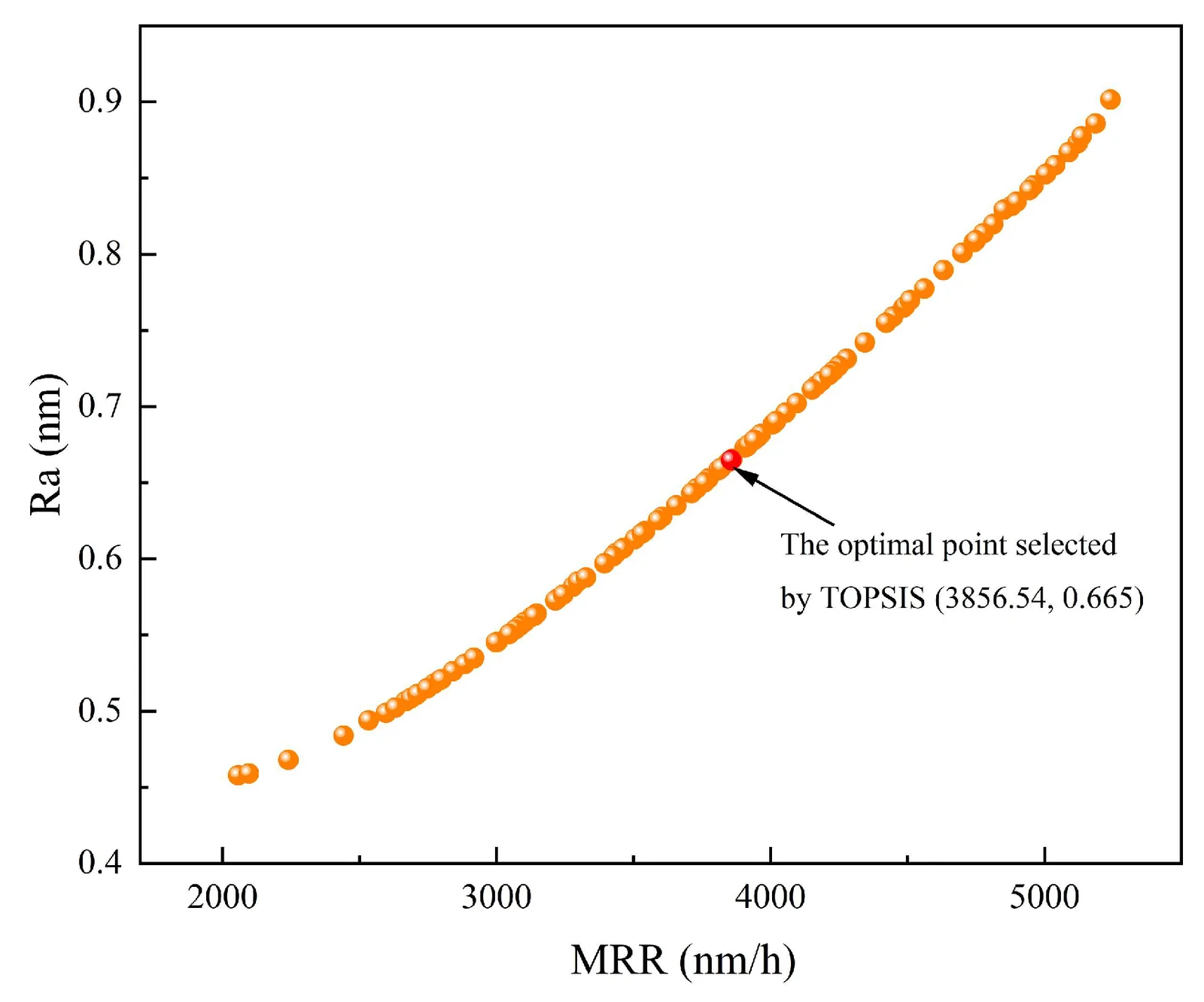
MRR–Ra Pareto front and compromise solution selected by TOPSIS.

**Table 1:** Factors and levels in the Box–Behnken design.

Experimental Parameters	Variable	Level		
		−1	0	1
H_2_O_2_ concentration (wt%)	*A*	5	10	15
Abrasive concentration (wt%)	*B*	0.25	0.75	1.25
Polishing speed (rpm)	*C*	40	50	60

Note: Concentrations are expressed as mass percentages in the prepared slurry.

**Table 2:** Box–Behnken design matrix and measured responses.

Run	*A* (H_2_O_2_, wt%)	*B* (Abrasive, wt%)	*C* (Speed, rpm)	MRR (nm/h)	Ra (nm)
1	15	1.25	50	5732.27	1.218
2	10	0.75	50	2095.67	0.523
3	10	0.25	40	1278.93	0.934
4	15	0.75	40	2018.62	0.912
5	5	0.75	60	1849.12	0.692
6	5	0.75	40	1540.93	0.861
7	10	0.75	50	2326.81	0.494
8	15	0.25	50	1479.30	0.901
9	10	0.75	50	1929.17	0.452
10	10	1.25	60	5100.49	0.922
11	5	1.25	50	4699.84	0.981
12	10	1.25	40	4299.20	1.121
13	15	0.75	60	2373.04	0.787
14	5	0.25	50	1171.11	0.873
15	10	0.25	60	1617.98	0.686

Runs 2, 7, and 9 are center-point runs.

**Table 3:** ANOVA for the MRR model.

Source	Degrees of Freedom	A_dj_SS	A_dj_MS	*F*	*p*	Significance
Model	9	31,880,000.00	3,542,000.00	54.14	0.0002	***
*A*	1	658,500.00	658,500.00	10.48	0.0230	**
*B*	1	25,510,000.00	25,510,000.00	389.87	0.0001	***
*C*	1	406,300.00	406,300.00	6.21	0.0550	*
*AB*	1	131,100.00	131,100.00	2.00	0.2160	
*AC*	1	534.30	534.30	0.0082	0.9315	
*BC*	1	53,416.45	53,416.45	0.8165	0.4076	
*A* ^2^	1	562.74	562.74	0.0086	0.9297	
*B* ^2^	1	4,808,000.00	4,808,000.00	73.49	0.0004	***
*C* ^2^	1	125,200.00	125,200.00	1.91	0.2251	
Error	5	327,100.00	65,420.79	2.07	0.3424	
Lack of fit	3	249,300.00	82,449.59			
Pure error	2	79,755.17	39,877.59			
Total	14	32,200,000.00				
*R*^2^ = 0.9898 *R*^2^_Adjusted_ = 0.9716						

Note: * *p* < 0.05, ** *p* < 0.01, and *** *p* < 0.001 indicate statistically significant differences.

**Table 4:** ANOVA for the Ra model.

Source	Degrees of Freedom	A_dj_SS	A_dj_MS	*F*	*p*	Significance
Model	9	0.6757	0.0751	51.81	0.0002	***
*A*	1	0.0211	0.0211	14.57	0.0124	**
*B*	1	0.0899	0.0899	62.03	0.0005	***
*C*	1	0.0686	0.0686	47.36	0.001	***
*AB*	1	0.0109	0.0109	7.53	0.0405	**
*AC*	1	0.000484	0.000484	0.33	0.5884	
*BC*	1	0.0006	0.0006	0.41	0.5482	
*A* ^2^	1	0.1483	0.1483	102.35	0.0002	***
*B* ^2^	1	0.3394	0.3394	234.19	0.0001	***
*C* ^2^	1	0.0558	0.0558	38.50	0.0016	***
Error	5	0.0072	0.00144	1.23	0.4781	
Lack of fit	3	0.0048	0.0016			
Pure error	2	0.0026	0.0013			
Total	14	0.6830				
*R*^2^ = 0.9894 *R*^2^_Adjusted_ = 0.9703						

Note: ** *p* < 0.01, and *** *p* < 0.001 indicate statistically significant differences.

**Table 5:** Comparison of the selected condition with benchmark design runs.

Condition	*A* (wt%)	*B* (wt%)	*C* (r/min)	MRR (nm/h)	Ra (nm)
Lowest measured Ra in the design, Run 9	10.00	0.75	50.00	1929.17	0.452
Highest measured MRR in the design, Run 1	15.00	1.25	50.00	5732.27	1.218
Selected compromise condition	9.79	1.08	54.33	3871.32	0.6747

Note: Values for Runs 9 and 1 are measured; values for the selected condition are model predictions.

**Table 6:** Experimental validation of the selected polishing condition.

Characterization	Predicted Value	Measured Value	Relative Deviation
MRR (nm/h)	3871.32	4283.79	10.65%
Ra (nm)	0.6747	0.712	5.53%

Note: The selected condition was 9.79 wt% H_2_O_2_, 1.08 wt% SiO_2_, and 54.33 rpm. Relative deviations were calculated using the unrounded predicted values.

## Data Availability

The original contributions presented in this study are included in the article. Further inquiries can be directed to the corresponding author.
